# Ammonia Volatilization Losses from Paddy Fields under Controlled Irrigation with Different Drainage Treatments

**DOI:** 10.1155/2014/417605

**Published:** 2014-03-11

**Authors:** Yupu He, Shihong Yang, Junzeng Xu, Yijiang Wang, Shizhang Peng

**Affiliations:** ^1^State Key Laboratory of Hydrology-Water Resources and Hydraulic Engineering, Hohai University, Nanjing 210098, China; ^2^Kunshan Water Conservancy Engineering Supervision of Quality and Safety and Water Conservancy Technology Extending Station, Kunshan 215300, China

## Abstract

The effect of controlled drainage (CD) on ammonia volatilization (AV) losses from paddy fields under controlled irrigation (CI) was investigated by managing water table control levels using a lysimeter. Three drainage treatments were implemented, namely, controlled water table depth 1 (CWT1), controlled water table depth 2 (CWT2), and controlled water table depth 3 (CWT3). As the water table control levels increased, irrigation water volumes in the CI paddy fields decreased. AV losses from paddy fields reduced due to the increases in water table control levels. Seasonal AV losses from CWT1, CWT2, and CWT3 were 59.8, 56.7, and 53.0 kg N ha^−1^, respectively. AV losses from CWT3 were 13.1% and 8.4% lower than those from CWT1 and CWT2, respectively. A significant difference in the seasonal AV losses was confirmed between CWT1 and CWT3. Less weekly AV losses followed by TF and PF were also observed as the water table control levels increased. The application of CD by increasing water table control levels to a suitable level could effectively reduce irrigation water volumes and AV losses from CI paddy fields. The combination of CI and CD may be a feasible water management method of reducing AV losses from paddy fields.

## 1. Introduction

In many countries, excess N is applied to farmlands to maximize grain yield. However, overapplication of N always results in low N use efficiency and serious N losses [1–4] and consequently leads to pollution of surface water, groundwater, and the atmosphere [5, 6]. In general, ammonia volatilization (AV) is the major pathway of N losses from farmlands [7]. In 1990, AV from fertilization was estimated to be 12.6 Tg N year^−1^ [8]. Aside from economic significance for the farmers, AV losses may have a negative ecological impact on atmospheric quality [9, 10]. Ammonia deposition from the atmosphere to land would cause soil acidification [11], promote eutrophication of surface water bodies, and affect terrestrial biodiversity [12]. Paddy fields for rice production are a source of NH_3_ to the atmosphere [13]. China is the primary rice producing country in the world. Its harvested area was 30.3 million hectares in 2011 [14]. Excess N fertilizer is applied to paddy soils in China [15], and urea is the commonly used N fertilizer. Extensive chemical N fertilizer application results in high AV losses from paddy fields in China [16, 17]. Therefore, studying reasonable strategies to reduce AV losses from paddy fields in China is of considerable importance.

Several water and fertilizer techniques may be useful for reducing AV losses from paddy fields [13, 16, 18, 19]. Controlled irrigation (CI) is a water management practice that has been shown to effectively reduce AV losses from paddy fields [19–21]. This practice is a widely adopted water-saving irrigation (WSI) method for rice cultivation in China. In CI fields, irrigation is applied only when the soil water content approaches the lower threshold for irrigation. No standing water is found after the regreening stage. Xu et al. [19] reported that seasonal AV losses from CI paddy fields were reduced by 14.0% compared with those from flooding irrigation (FI) paddy fields. Xiao et al. [22] also argued that total amounts and loss rate of AV were lower in CI compared with FI.

Controlled drainage (CD; also called drainage water management) emerged as an effective method for reducing losses of N in drainage waters and has been tested in the United States [23, 24], Canada [25, 26], Sweden [27], and China [28]. This method is typically applied by installing a structure in the subsurface drain to manage the groundwater table. Variations of groundwater table could influence N and soil water dynamics in root zone [27, 29], which may affect AV losses from farmlands. However, no study has focused on the effect of CD on AV losses. In addition, CI has been confirmed as an effective way to reduce AV losses from paddy fields. However, whether the combination of CI and CD can reduce AV losses from paddy fields has yet to be determined. Recognizing the above concerns, in this study, the effect of CD on AV losses from CI paddy fields was investigated by managing water table control levels with the use of a lysimeter equipped with an automatic water table control system.

## 2. Materials and Methods

### 2.1. Experimental Site

Experiments were conducted in lysimeters at the Kunshan Experiment Station in Suzhou, Jiangsu Province (31° 15′ 50′′ N; 120° 57′ 43′′ E), which is located in the lower reach of the Taihu Lake Basin. The study area has a subtropical monsoon climate with an average annual temperature of 15.5°C, annual precipitation of 1,097.1 mm, and annual evaporation of 1,365.9 mm. The soil type of the experimental field is dark-yellow hydromorphic paddy soil. The soil texture in the plowed layer is clay with 21.88 g kg^−1^ of organic matter, 1.03 g kg^−1^ of total N, 1.35 g kg^−1^ of total P, 20.86 g kg^−1^ of total K, and pH 7.4 (soil/water, 1 : 2.5). The bulk density of soil in the plowed layer is 1.24 g cm^−3^. The saturated soil water contents (vol vol^−1^) for the layers of 0–20 cm, 0–30 cm, and 0–40 cm are 52.0%, 50.1%, and 47.9%, respectively.

### 2.2. Experimental Design

The experiment consisted of three drainage treatments, namely, controlled water table depth 1 (CWT1), controlled water table depth 2 (CWT2), and controlled water table depth 3 (CWT3). Every treatment was conducted in triplicate in nine lysimeters.

**Figure 1:**
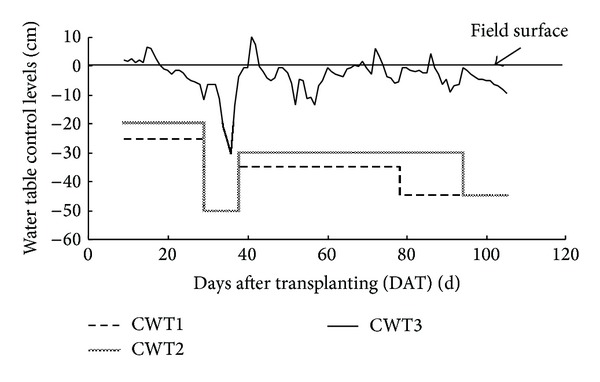
Water table control levels for CWT1, CWT2, and CWT3.

For CWT1, the water table control levels in different stages were selected based on previous studies on increasing rice yields in paddy fields of Southeast China [30]. The water table control levels in CWT2 were controlled based on the rice root zone depths in different stages according to the water table management that was tested in the humid regions of Eastern Canada and Midwestern United States [31]. The water table control levels in the later tillering stage and milk stage were also adjusted depending on the characteristics of rice growth and cultivation needs. For CWT3, the water table control levels were adjusted daily based on the actual water table depths that were measured by using a water table observation well, which was installed in the open paddy fields outside the lysimeter.  Figure 1 presented the water table control levels in different stages for CWT1, CWT2, and CWT3.

The rice variety is Japonica Rice Jia 04-33. The rice seedlings were transplanted on June 28, 2012. Three to four plants were transplanted in every hill and were harvested on October 24, 2012. The fertilization process conducted in this experiment followed local rice cultivation practices (Table 1). The irrigation management employed for all plots was CI. A 5 mm to 25 mm standing water depth was maintained during the regreening stage for CI; irrigation was controlled by root zone soil water content, and standing water was avoided in other stages except during pesticide and fertilizer applications [19].

### 2.3. Experimental Layout

Experiments were conducted in nine lysimeters with a mobile shelter and gallery. Each lysimeter had an area of 2.5 m × 2 m and a depth of 1.3 m. The influence of rainfall was avoided by using the mobile shelter to accurately regulate soil moisture in all treatments. Each lysimeter was individually irrigated by using a pipe installed with a water meter. Water leakage from each plot was drained through a water-permeable tube (40 mm in inner diameter) installed 1.2 m below the soil surface into the gallery.

**Figure 2:**
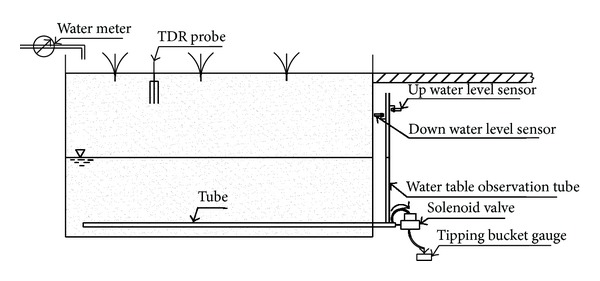
Automatic water table control system.

Irrigation was applied only when the observed pond water depth or soil moisture approached the lower threshold for irrigation. Subsurface drainage was conducted based on the water table control levels (Table 1) by using an automatic water table control system, which was installed on each drain tube in the gallery (Figure 2). A transparent polymethyl methacrylate tube connected to the drain tube was used to observe the water table in the plot. The water table signal was sensed by two moveable water level sensors (FKC1810-N, Jiazhun) connected to the water table observation tube. This system controlled the drainage by switching a solenoid valve (SLP-15, Wankong) based on the signal sent by the water level sensors. The solenoid valve was opened for drainage when the up water level sensor sensed the water table signal. The water table decreased during the drainage. The solenoid valve was closed to stop drainage when the down water level sensor lost the water table signal. Water leakage volumes were measured by using an automatic tipping bucket gauge placed at the end of the system. Two water level sensors were placed 2 cm above and below the water table control levels. The different drainage treatments were implemented by properly changing the positions of the water level sensors according to their water table control levels.

### 2.4. Field Measurement and Sampling

Soil moistures were measured daily by using a Trease system (6050X3, SEC) when no pond water remained in the paddy fields. Pond water depths in the paddy fields were measured daily using a vertical ruler. Irrigation water volumes were recorded by using a water meter installed on the pipe of each plot. The water leakage volumes were measured by using the tipping bucket gauge (0.05 mm resolution). The obtained data were then transferred to a computer.

AV rate from the paddy fields was measured in triplicate via ventilation method using 20 cm high PVC collectors with a phosphoglycerol-soaked sponge as an absorbent [32]. Samples were collected daily after N application for 1 week, then at a 3 d interval for another week, and finally at a 1week interval. The samples collected in the phosphoglycerol-soaked sponge from the paddy fields by the PVC collectors were immediately immersed in 450 mL of 1.0 mol L^−1^ KCl solution in 500 mL containers and were shaken on a reciprocating shaker. Then, NH_4_^+^–N concentrations in the extract solutions extracted by the AV loss collectors were analyzed by using the indophenol blue method [33] with an ultraviolet-visible spectrophotometer (UV-2800, UNICO). The AV rate was calculated by using the following equation and seasonal AV losses during rice growth stage were summed:

(1) $$\begin{array}{c} R_{\text{AV}}=\frac{M}{A\cdot D}\times 10^{-2}, \end{array}$$

where *R*_AV_ is the AV rate (kg N ha^−1^ d^−1^), *M* is the ammonia N collected by the PVC collector (mg), *A* is the cross-sectional area of the PVC collector (m^2^), and *D* is the interval for AV sample collection (d).

### 2.5. Chemical and Statistical Analysis

Statistical analysis was carried out following standard procedures on a randomized plot design (SPSS 17.0). Significance was calculated based on least significant difference (LSD) test at the 0.05 probability level.

## 3. Results

### 3.1. Water Management Regimes

**Figure 3:**
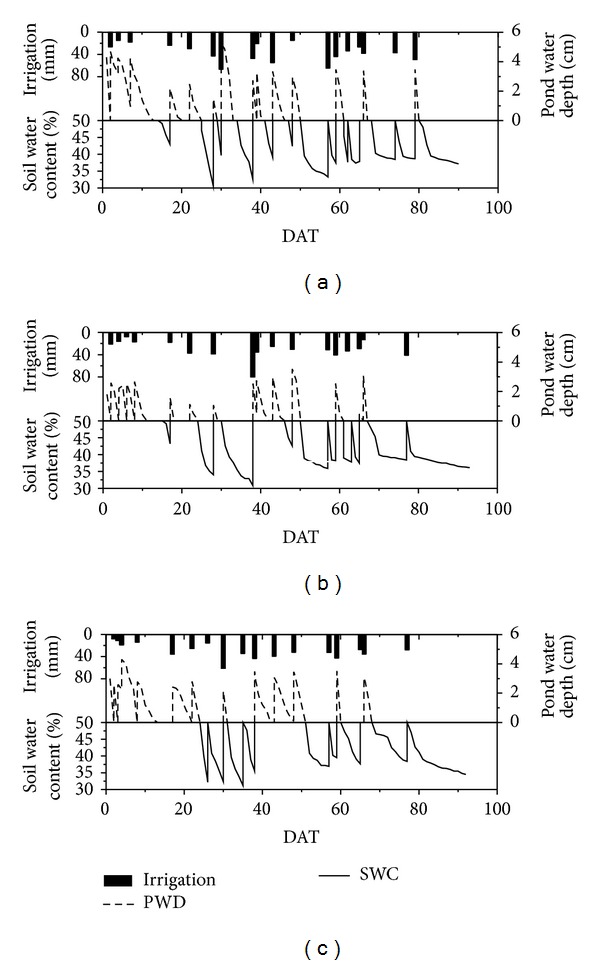
Typical pond water depth, soil water content, and irrigation for CWT1, CWT2, and CWT3.

As the water table control levels increased, less wet-dry cycles and irrigation water volumes were observed in the CI paddy fields. CWT1, CWT2, and CWT3 had 13, 10, and 9 wet-dry cycles, respectively (Figure 3). After the regreening stage, CD was implemented in the CI paddy fields. From the initial tillering stage to harvest, fields were irrigated 15, 14, and 14 times in CWT1, CWT2, and CWT3, respectively, along with pesticide and fertilizer applications. The irrigation water volumes of CWT2 and CWT3 were 481.3 and 465.1 mm, which indicate an 18.6% and 21.3% decrease, respectively, compared with CWT1 (591.3 mm). The irrigation water volume of CWT3 was 3.4% lower than that of CWT2. With the increase in water table control levels, more water was stored in paddy fields after irrigation rather than that drained. Water storage in paddy soil increased, which potentially increased the water supply from shallow groundwater to the root zone soil and lengthened the duration of soil moisture depletion to the lower threshold for irrigation. Therefore, the wet-dry cycles and irrigation water volumes of paddy fields decreased following the increase in the water table control levels.

Experiments were carried out in lysimeters with shelter; irrigation times and volumes were much higher than those in open fields. Irrigation water demands were calculated by subtracting the seasonal effective rainfall from total irrigation water volumes. Seasonal effective rainfall was calculated as 303.5 mm. Irrigation water demands in CWT1, CWT2, and CWT3 were 287.8, 177.8, and 161.6 mm, respectively. The irrigation water volumes were lower than those in freely drained paddy fields under WSI method. The irrigation water volume obtained by the System of Rice Intensification (SRI) method reported by Choi et al. [34] was 243.2 mm. A similar result was noted by Cabangon et al. [35] who observed that the irrigation water volume ranged from 203 mm to 339 mm in alternate wetting and drying irrigation (AWD) for middle rice. The respective results reported by Xu et al. [20] and Li et al. [16] were 315.0 mm to 328.2 mm and 480.0 mm to 640.0 mm, which were obtained by using the single CI and zero drainage practice, respectively.

### 3.2. Ammonia Volatilization Rate

**Figure 4:**
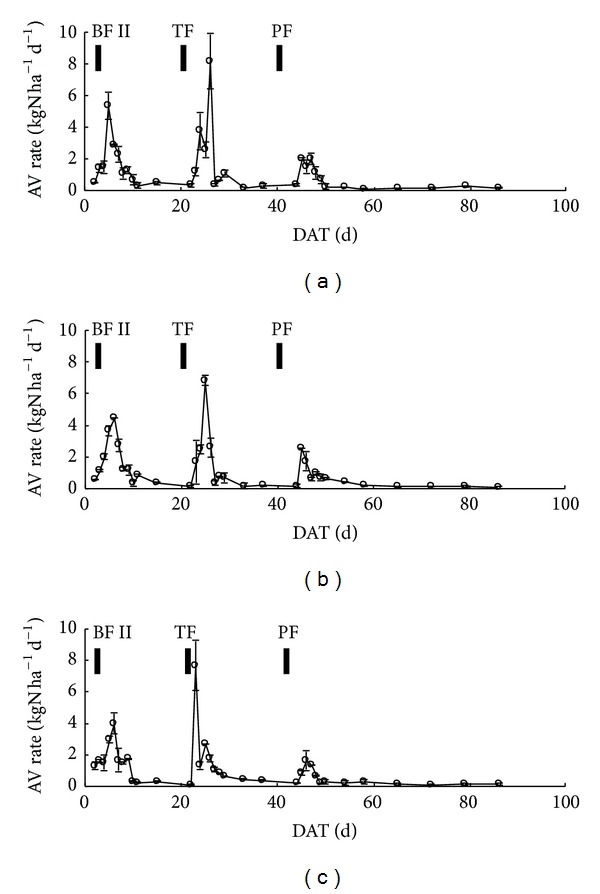
Ammonia volatilization rates from (a) CWT1, (b) CWT2, and (c) CWT3.

The pattern of AV rates from paddy fields was similar to those in CWT1, CWT2, and CWT3 treatments (Figure 4). N application was the predominant factor in AV rate from paddy fields, as AV rate always peaked 1 d to 3 d after top-dressing fertilization and then decreased to low values within 10 d. AV rates increased to 6.244, 4.491, and 3.988 kg N ha^−1^ d^−1^ on 6 DAT, 5 DAT, and 5 DAT in CWT1, CWT2, and CWT3, respectively, after the use of base fertilizer II (BF II) on 4 DAT. Tillering fertilizer (TF) on 22 DAT led to higher peaks of 8.178, 6.835, and 7.683 kg N ha^−1^ d^−1^ on 26 DAT, 25 DAT, and 23 DAT in CWT1, CWT2, and CWT3, respectively. Panicle fertilizer (PF) also resulted in high AV rates 3 d after fertilization. However, the AV rate peaks after PF were much lower than those after BF II and TF due to lower N and higher canopy cover.

As the water table control levels increased, lower amounts of AV rate peak were observed in the paddy fields; 7, 6, and 5 AV rate peaks were observed in CWT1, CWT2, and CWT3, respectively. AV rate always peaked again following the absence or reemergence of shallow water during one week after N application. For example, after TF on 22 DAT, AV rate in CWT1 first peaked at 3.762 kg N ha^−1^ d^−1^ on 24 DAT with 6 mm pond water and decreased to 2.556 kg N ha^−1^ d^−1^ on 25 DAT when the pond water disappeared. AV rate then increased and reached its higher peak of 8.178 kg N ha^−1^ d^−1^ on 26 DAT after the absence of shallow water. The reemergence of shallow water also induced strong AV rates from paddy fields. AV rate in CWT2 after TF first peaked at 6.835 kg N ha^−1^ d^−1^ on 25 DAT and reduced rapidly to 0.363 kg N ha^−1^ d^−1^ on 27 DAT when soil water content decreased to 35.1%. It was irrigated on 28 DAT when the soil water content was depleted to 34.0%. After irrigation, shallow pond water (11.0 mm) remained in CWT2. Then, the second AV rate peak in CWT2 appeared on 28 DAT in the reemergence of shallow water due to reflooding.

### 3.3. Seasonal Nitrogen Loss by Ammonia Volatilization

Increases in water table control levels reduced AV losses from paddy fields. Seasonal AV losses from CWT1, CWT2, and CWT3 were calculated to be 59.8, 56.7, and 53.0 kg N ha^−1^, which account for 18.8%, 17.8%, and 16.3% of applied N, respectively (Table 2). AV losses from CWT3 were the lowest, which were 13.1% and 8.4% lower than those from CWT1 and CWT2, respectively. A significant difference in the seasonal AV losses was confirmed between CWT1 and CWT3. CWT2 resulted in a 5.1% decrease in AV losses compared with CWT1. Weekly AV losses followed by fertilization were highest and lowest after TF and PF among treatments, respectively, due to the difference of N amounts (121.8 kg N ha^−1^ TF versus 87.0 kg N ha^−1^ PF). The weekly AV losses after BF II also remained at high values and were a little lower than those after TF. Considering that N in BF II was lower, the ratios of weekly AV losses to applied N after BF II were higher than those of TF and PF.

After the regreening stage, CD was implemented in the CI paddy fields. Lower weekly AV losses followed by TF and PF were observed as the water table control levels increased. Weekly AV losses followed by TF from CWT3 were relatively low and similar to that from CWT2. The weekly AV losses followed by TF from CWT2 and CWT3 were 12.7% and 9.3% lower than that from CWT1, respectively. Significant differences in the weekly AV losses followed by PF were confirmed among the treatments, with lower values for CWT3 (Table 2). The weekly AV losses followed by PF from CWT3 were 35.1% and 29.3% lower than those from CWT1 and CWT2, respectively. CWT2 exhibited an 8.2% decrease in weekly AV losses after PF compared with CWT1.

## 4. Discussion

In this experiment, the absence of shallow water may induce short-term strong AV rates from CI paddy fields among different drainage treatments; for example, the absence of shallow water on 26 DAT in CWT1 enhanced AV rates. This result confirmed that an extremely high flux of NH_3_ volatilization was observed in the absence of a water table [36]. In addition, Zhao et al. [37] suggested that SRI paddy kept in a moist condition without standing water would enhance AV. In our experiment, the soil in the absence of shallow water was similar to the moist soil under SRI. The absence of shallow water may increase NH_4_^+^–N concentrations in soil and soil solution, thereby enhancing the AV rates.

Previous studies indicated that a high floodwater level could prevent AV loss due to the dilution effect on NH_4_^+^–N concentrations in floodwater [38, 39], while the shallow water after reflooding enhanced AV rates from CI paddy fields. For example, the reemergence of shallow water on 28 DAT in CWT2 enhanced AV rates, which confirmed that relatively shallow water was likely to induce strong AV rates due to higher NH_4_^+^–N concentrations and higher temperatures [36, 40]. The standing water after irrigation was avoided in CI except during pesticide and fertilizer applications. Shallow water remained after reflooding due to the soil water content depletion to the lower threshold. In addition, the reflooding disturbed the paddy soils, which may release the NH_4_^+^–N absorbed by surface soil into floodwater [41]. As a result, the reemergence of shallow water after reflooding enhanced AV rates from CI paddy fields. In contrast, Zhu et al. [42] found that reflooding of paddy fields after applying urea reduced ammonia loss. But, the depth and duration of pond water after the reflooding in the experiment of Zhu et al. [42] were much higher and longer than those in our experiment. Because of the higher floodwater level, ammonia loss in the experiment of Zhu decreased after reflooding.

As the water table control levels increased, less times of absence and reemergence of shallow water were observed in the CI paddy fields during the rice growth season, especially during the first week after N application. The times of reemergence of shallow water during the first week followed by TF and PF were the same among different treatments, which may be influenced by the reflooding for pesticide application on 48 DAT. The increases in water table control levels resulted in less times of absence of shallow water during the first week after N application and reduced AV rates. The average water table control levels in CWT3 during the first week after PF were −2.9 cm, which were much higher than those in CWT1 and CWT2 (−35.0 and −30.0 cm, resp.). The higher water table control levels in CWT3 resulted in less water leakage and delayed the absence of shallow water. Hence, CWT3 had significantly restrained AV rates for avoiding the absence of shallow water during the first week after PF compared with CWT1 and CWT2, whereas increases in water table control levels had no effect on the absence of shallow water a week after TF. No significant differences in weekly AV losses followed by TF among treatments were confirmed. The average water table control levels in CWT1, CWT2, and CWT3 were −25.0, −20.0, and −4.9 cm, respectively. The differences of water table control levels among treatments were lower than those after PF, which may be not high enough to affect the absence of shallow water and AV rates.

During other periods, as water table control levels increased, AV rates also decreased due to less times of absence and reemergence of shallow water. For example, from 58 DAT to 86 DAT, AV losses from CWT1, CWT2, and CWT3 were 4.62, 4.02, and 3.72 kg N ha^−1^, respectively, and times of absence and reemergence of shallow water were 6, 5, and 4, respectively. Thus, we could confirm that increasing water table control levels to a suitable level during the rice growth season, especially during the first week after N application, could be helpful in reducing AV rates from CI paddy fields. In addition, increasing water table control levels also reduced leaching risks of N [26, 27].

## 5. Conclusions

The effects of CD on AV losses from CI paddy fields are obvious. The application of CD by increasing water table control level to a suitable level could effectively reduce AV losses and irrigation water volumes from CI paddy fields. The combination of CI and CD may be a feasible water management method of reducing AV losses from paddy fields.

AV rate always peaked again following the absence or reemergence of shallow water. The increases in water table control levels resulted in less times of absence and reemergence of shallow water in the paddy fields during the rice growth season, especially during the first week after N application. AV losses from paddy fields reduced due to decreases in the times of absence and reemergence of shallow water. Seasonal AV losses from CWT3 were 13.1% and 8.4% lower than those from CWT1 and CWT2, respectively, and a significant difference in the seasonal AV losses was confirmed between CWT1 and CWT3. Less weekly AV losses followed by TF and PF were also observed as the water table control levels increased. As the water table control levels increased, the irrigation water volumes of CWT2 and CWT3 were 18.6% and 21.3% lower than that of CWT1, respectively. The irrigation water volume of CWT3 was 3.4% lower than that of CWT2.

## Figures and Tables

**Table 1:** Time and amount of fertilization.

	Time (month-day)	DAT (d)	N (kg ha^−1^)	P_2_O_5_ (kg ha^−1^)	K_2_O (kg ha^−1^)
Base fertilizer I	6-27		45	45	45
Base fertilizer II	7-2	4	64.5		
Tillering fertilizer	7-20	22	121.8		
Panicle fertilizer	8-10	43	87		

Total			318.3	45	45

**Table 2:** Ammonia volatilization losses from CWT1, CWT2, and CWT3.

Treatment	AV losses (kg N ha^−1^)				Ratio of AV losses to applied *N* (%)			
	During a week after fertilization			Seasonal	During a week after fertilization			Seasonal
	BF II	TF	PF		BF II	TF	PF	
CWT1	14.5 (0.4)^ab^	17.4 (1.1)^a^	7.6 (0.1)^a^	59.8 (2.5)^a^	22.4 (0.6)^ab^	14.2 (0.9)^a^	8.7 (0.1)^a^	18.8 (0.8)^a^
CWT2	15.1 (0.4)^a^	15.2 (2.1)^a^	7.0 (1.1)^a^	56.7 (3.1)^ab^	23.4 (0.6)^a^	12.4 (1.8)^a^	8.0 (1.3)^a^	17.8 (1.0)^ab^
CWT3	13.0 (1.5)^b^	15.7 (1.6)^a^	4.9 (0.7)^b^	53.0 (1.3)^b^	20.2 (2.4)^b^	12.9 (1.3)^a^	5.7 (0.8)^b^	16.3 (0.4)^b^

Means in the same column followed by the same letter are not significantly different (*P* < 0.05) by LSD.

Numbers in parenthesis are standard deviation.
